# Metabolites in the regulatory risk assessment of pesticides in the EU

**DOI:** 10.3389/ftox.2023.1304885

**Published:** 2023-12-19

**Authors:** Olavi Pelkonen, Khaled Abass, Juan Manuel Parra Morte, Martina Panzarea, Emanuela Testai, Serge Rudaz, Jochem Louisse, Ursula Gundert-Remy, Gerrit Wolterink, Dorne Jean-Lou CM, Sandra Coecke, Camilla Bernasconi

**Affiliations:** ^1^ Research Unit of Biomedicine, Pharmacology and Toxicology, University of Oulu, Oulu, Finland; ^2^ Department of Environmental Health Sciences, College of Health Sciences, University of Sharjah, Sharjah, United Arab Emirates; ^3^ Sharjah Institute for Medical Research (SIMR), University of Sharjah, Sharjah, United Arab Emirates; ^4^ Research Unit of Biomedicine and Internal Medicine, Faculty of Medicine, University of Oulu, Oulu, Finland; ^5^ EFSA, European Food Safety Authority, Parma, Italy; ^6^ Mechanisms, Biomarkers and Models Unit, Environment and Health Department, Istituto Superiore di Sanità, Rome, Italy; ^7^ School of Pharmaceutical Sciences, University of Geneva, CMU, Geneva, Switzerland; ^8^ Wageningen Food Safety Research (WFSR), Wageningen, Netherlands; ^9^ Institute of Clinical Pharmacology and Toxicology, Charité–Universitätsmedizin Berlin, Freie Universität Berlin, Humboldt-Universität zu Berlin, Berlin, Germany; ^10^ Centre for Prevention, Lifestyle and Health, National Institute for Public Health and the Environment (RIVM), Bilthoven, Netherlands; ^11^ European Commission, Joint Research Centre (JRC), Ispra, Italy

**Keywords:** xenobiotic metabolism, pesticide metabolite, risk assessment, *in vitro*/*in silico* testing, analytical methods, unique human metabolite, disproportionate human metabolite

## Abstract

A large majority of chemicals is converted into metabolites through xenobiotic-metabolising enzymes. Metabolites may present a spectrum of characteristics varying from similar to vastly different compared with the parent compound in terms of both toxicokinetics and toxicodynamics. In the pesticide arena, the role of metabolism and metabolites is increasingly recognised as a significant factor particularly for the design and interpretation of mammalian toxicological studies and in the toxicity assessment of pesticide/metabolite-associated issues for hazard characterization and risk assessment purposes, including the role of metabolites as parts in various residues in ecotoxicological adversities. This is of particular relevance to pesticide metabolites that are unique to humans in comparison with metabolites found in *in vitro* or *in vivo* animal studies, but also to disproportionate metabolites (quantitative differences) between humans and mammalian species. Presence of unique or disproportionate metabolites may underlie potential toxicological concerns. This review aims to present the current state-of-the-art of comparative metabolism and metabolites in pesticide research for hazard and risk assessment, including One Health perspectives, and future research needs based on the experiences gained at the European Food Safety Authority.

## Introduction

Metabolite is a scientific concept defined in many ways (see Box 1). On the basis of the different definitions, one can conclude that the meaning of “metabolite” is context-dependent. In brief, it needs specifying attributes, e.g., referencing to the parent compound such as drug metabolite, pesticide metabolite, or to the metabolic pathway described as intermediary metabolite. In pharmacology and toxicology, metabolites often refer to the reaction products of xenobiotic metabolism, suggesting biochemical pathways. However, products from synthetic molecules produced by physicochemical forces, i.e., not by processes of living organisms, are also often called “metabolites” (also the expression ‘transformation product’ is being used). To further add complexity to these definitions, consider the example “liver (or hepatic) metabolite”. It may mean a metabolite of an endogenous substance, originating typically in the liver, or it may mean a metabolite produced/formed in the liver from an exogenous substance, i.e., drug, pesticide or any other kind of contaminants or chemical by xenobiotic-metabolizing enzymes. In this article, the term “metabolite” is used as depicting compounds derived e.g., from a pesticide via the action of xenobiotic-metabolizing enzymes, either as primary metabolites, or more distal, secondary metabolites if successive biotransformation has taken place on primary or more distal metabolites.

BOX 1 **Definitions of “a metabolite” (checked on 04.07.2023).**

Wikipedia: In biochemistry, a metabolite is intermediate or end product of metabolism.

Merriam-Webster: 1: metabolite is a product of metabolism; 2: a substance essential to the metabolism of a particular organism or to a particular metabolic process.

Cambridge dictionary: any substance involved in metabolism (= the chemical processes in the body needed for life).

Oxford lexico: In biochemistry, a substance formed in or necessary for metabolism.

FDA: A compound derived from the parent drug through phase 1 and/or phase 2 metabolic pathways.

NCI: A substance made or used when the body breaks down food, drugs or chemicals, or its own tissue (for example, fat or muscle tissue). This process, called metabolism, makes energy and the materials needed for growth, reproduction, and maintaining health. It also helps get rid of toxic substances.

NIH-NLM: A metabolite is any substance produced during metabolism (digestion or other bodily chemical processes). The term metabolite may also refer to the product that remains after a drug is broken down (metabolized) by the body.

In pharmacology and drug development, the metabolite pattern originating from a drug is of high significance for several reasons: drugs are administered purposefully in high enough doses for desired actions in the body (e.g., to ensure ‘efficacy’): consequently, metabolism of a drug active *per se* should be evaluated for effective drug treatment (too quick clearance would limit or avoid the therapeutic action) or in case of pro-drugs, the effective metabolite(s) should be formed in sufficient doses; at the same time metabolites should be evaluated for safe drug treatment and for potential metabolite-associated adverse effects, with a special and necessary focus on quantitative aspects of kinetic and dynamic characteristics. Furthermore, current regulations require that residues of human and veterinary pharmaceutical products should be monitored in the environment, in particular in surface water and in food, which means a potentially analogous situation with regards to pesticides, i.e., exposure of humans and living organisms via the environment (Heberer, 2002; EMA, 2018). Although pesticides are not administered purposefully to humans, humans and other living organisms are exposed to pesticides and their metabolites via food/feed or through the environment, albeit at highly variable, but generally low amounts. It is good to remember that at present most pesticides are small-molecular drug-like entities for which it is necessary to assess the identity and possible adverse effects of metabolites.

The current drive is to develop comprehensive methods to assess the exposome, defined as a collection of environmental factors, such as stress and diet, to which an individual is exposed, and which can influence health (Collins English Dictionary). In toxicology the exposome generally equates to exposure of an organism to “all foreign chemicals” (see, e.g., Olesti et al., 2021; Manz et al., 2023). This definition also anticipates that potential metabolites of any pesticide should be determined and characterized to trace the origins of “foreign chemicals” in the exposome. The exposome concept itself implicates as default “real-life” situations and it could be envisaged that in the end there is a need to trace any exposome constituent to an actual exposing chemical entity (see e.g., Govarts et al., 2023). Furthermore, a more integrative assessment of human, animal, and environmental exposure and health effects is required to tackle the challenges described in the One Health concept (https://www.cdc.gov/onehealth/basics/index.html).

The principal aim of this review is to present the current situation and some future outlooks of the significance of pesticide metabolism and metabolites in toxicological hazard and risk assessment activities, based on EFSA and EU experiences. First, as a background, general features and relevance of xenobiotic metabolism and metabolites are presented, and a more detailed description of current and future regulatory work.

## Pesticide metabolites–relevance to hazard and risk assessment

The European Union (EU) pesticides database contains 1,478 approved and non-approved individual chemical entities (active substances). The approved active substances can be used as formulations, called plant protection products (PPP), authorised by Member States (EC, 2022). From the regulatory point of view, a pesticide active substance is extensively assessed (see Commission Regulation (EU) No 284/2013 setting out the data requirements for plant protection products, in accordance with Regulation (EC) No 1107/2009 (EUR-Lex - 32013R0283 - EN - EUR-Lex (europa.eu)). Plant protection products, besides the pesticide active substance, are composed of a variable number of other chemicals, including solvents, adjuvants, emulsifiers and many other so called inert ingredients (US-EPA, 1998). Data requirements for the regulatory assessment of such formulations are provided in Commission Regulation (EU) No 284/2014 (Section 7), in accordance with the above mentioned EC Regulation (EUR-Lex - 32013R0284 - EN - EUR-Lex (europa.eu)). It is possible that the additional substances in formulations may affect the kinetics and dynamics of an active ingredient, which is a topic that is not extensively studied thus far (Braeuning and Marx-Stoelting, 2021), unless PPP are tested as a whole mixture.

The fate of a xenobiotic within an organism, be it rat or a human being, describing how and at what extent the chemical entity is metabolized and eliminated, can greatly impact its potential for toxicity. With regards to the risk assessment of pesticides, a major issue is to identify those metabolites which are expected or predicted to present serious hazards to humans or other living organisms. A major consideration in hazard and risk assessment is potential species differences in metabolism. To be able to confidently apply findings from any kind of animal toxicological study to humans, it is vital to determine whether humans are exposed to the same parent compound(s) and/or related metabolites as those that are present in the test species. This understanding is critical in ensuring the validity of extrapolating study data to real-world scenarios.

### Toxicokinetics

Toxicokinetics examines the fate of a xenobiotic as it enters, moves through, and exits the body, and is divided into the processes of absorption, distribution, metabolism, and excretion (ADME). As an example, Abass et al. (2021) published recently a review on toxicokinetic and toxicodynamic properties of chloro-s-triazine pesticides. Among these processes, metabolism is particularly significant as it can greatly affect the overall toxicity profile of a compound. In the first step of metabolism, known as phase I, enzymes, typically the cytochrome P450 (CYP) enzyme system but also other enzyme systems (e.g., flavin monooxygenases, peroxidases, amine oxidases, dehydrogenases, xanthine oxidases), biotransform the chemical through, e.g., oxidation, reduction, de-alkylation, etc., and often make the compound more water soluble, adding or unmasking functional groups making the primary metabolite suitable for the next biotransformation step. In addition to the oxidative reactions there are different types of hydrolytic reactions catalysed by enzymes like carboxylesterases and epoxide hydrolases. Next, in phase II, conjugating enzyme systems add endogenous substrates such as glucuronic acid, sulfuric acid, acetic acid, or an amino acid and convert the substance into a more water-soluble and excretable form. Usually, these enzymatic reactions are beneficial as they aid in eliminating foreign compounds. However, in certain cases, these enzymes can transform a substance into a reactive form, a phenomenon known as metabolic (bio)activation.

### Metabolism

Some pesticides are not metabolized to a significant extent in mammals and are excreted as such. For example, for glyphosate, no genuine human or rat metabolite has been observed, whereas some plants and bacteria convert glyphosate to aminomethylphosphonic acid (AMPA) and perhaps to other related or more distal products (Singh et al., 2020). For certain structurally related groups of pesticides, there exist common metabolites creating additional considerations and difficulties in exposure and regulations. Such pesticide groups are carbamates (see discussion below) and pyrethroids (EFSA PPR Panel et al., 2022). For a vast majority of pesticides, variable numbers and quantities of metabolites have been detected in humans and other mammals (for rat examples, see Table 1), as well as in the environment. It is obvious that this complexity leads to difficulties in analytical capabilities and a cutoff point of 5% out of the administered dose of the parent has been set for elucidating the identity of an individual metabolite, as in OECD TG417. However, it is important to remember that quantity as such, be it relative or absolute, does not necessarily imply toxicity hazard or toxic potency of a pesticide or its metabolites.

**TABLE 1:** Some basic toxicokinetic characteristics of example pesticides based on the regulatory rat *in vivo* toxicokinetic study. The data has been collected from assessment and regulatory documents.

	Bifenthrin^1^	Isoflucypram^2^	Terbuthylazine^3^
Absorption	50% absorption via oral route in rat in 4–6 h	84%–88% (based on urinary (2.4%–5.9%) and biliary (78%–85%) excretion within 48 h) (based on available data, single dose administration in bile-cannulated rats at 2 mg/kg bw) (100% oral absorption considered appropriate for the AOEL and AAOEL; 50% post-hepatic systemic availability)	Rapid, 79% following low dose administration in females based on urinary and biliary excretion, cagewash and carcass residues 48 h after administration
Distribution	Fat and skin mainly (3% of the dose remains in tissues)	Widely distributed (highest level in liver)	Widely distributed; initial distribution into fat. Significant and persistent binding to blood cells
Metabolism	Via hydrolysis, oxidation and conjugation. No preferential enantiomeric absorption, biotransformation or elimination of bifenthrin S-and R-enantiomers. No main metabolites, all less than 10%	Extensively metabolised (>95%); no major metabolite (i.e., >10% of the administered dose) in urine; main metabolites in plasma	Extensive metabolism in the rat; only trace level of unchanged terbuthylazine detected
		N-demethylation of the pyrazole methyl and/or oxidation of the isopropyl group to desmethyl carboxylates or lactate followed by glucuronidation	
Excretion	Elimination complete within 48 h urine (13%–25%) and faeces (63%–88%), 3% remained in tissues and organs	Rapid and extensive (>90% within 48 h), mainly via bile (in bile duct cannulated animals: 78%–85% within 48 h via bile, 16%–21% via faeces, 2.4%–5.9% via urine)	Rapid excretion: 60%–65% in urine and 30%–40% in faeces within 96 h (most of it during the first 48 h). Biliary excretion within 48 h: 40%–64% (in females and males respectively)
Estimation of half-life or clearance	not possible to estimate	not possible to estimate	not possible to estimate

1 https://efsa.onlinelibrary.wiley.com/doi/epdf/10.2903/j.efsa.2011.2159—Appendix A (List of end points for the active substance and the representative formulation).

2 https://efsa.onlinelibrary.wiley.com/doi/10.2903/j.efsa.2022.7328—Appendix B (List of end points for the active substance and the representative formulation).

3 https://efsa.onlinelibrary.wiley.com/doi/epdf/10.2903/j.efsa.2011.1969—Appendix A (List of end points for the active substance and the representative formulation).

Most pesticides are metabolised along complex pathways involving hepatic and non-hepatic phase I and II xenobiotic-metabolizing enzymes, and also other principally endogenous substance-transforming enzymes, resulting in highly variable numbers and amounts of metabolites (see examples in Table 1). Furthermore, one has not to forget the role of gut (and skin etc.) microbes in pesticide metabolism and actions (Meng et al., 2020). Characterising the enzymatic basis of such complex pathways is of considerable importance because metabolising enzymes determine rate and extent of various pathways and ultimately are responsible for the metabolic clearance of metabolizable pesticides and metabolites (of course, urinary and biliary excretion are significant, depending on the pesticide). As an example, at least one human recombinant CYP enzyme was involved in the *in vitro* metabolism of selected 63 different pesticides, resulting in a total of 495 CYP-associated metabolic reactions (Abass et al., 2012). Thus, one single CYP can metabolise a high number of different pesticides with variable efficiency, as well as a single pesticide can be metabolized by many different CYP isoforms with different affinity and overall clearance.

### Carbamates: metabolism and species differences as an example

**FIGURE 1:**
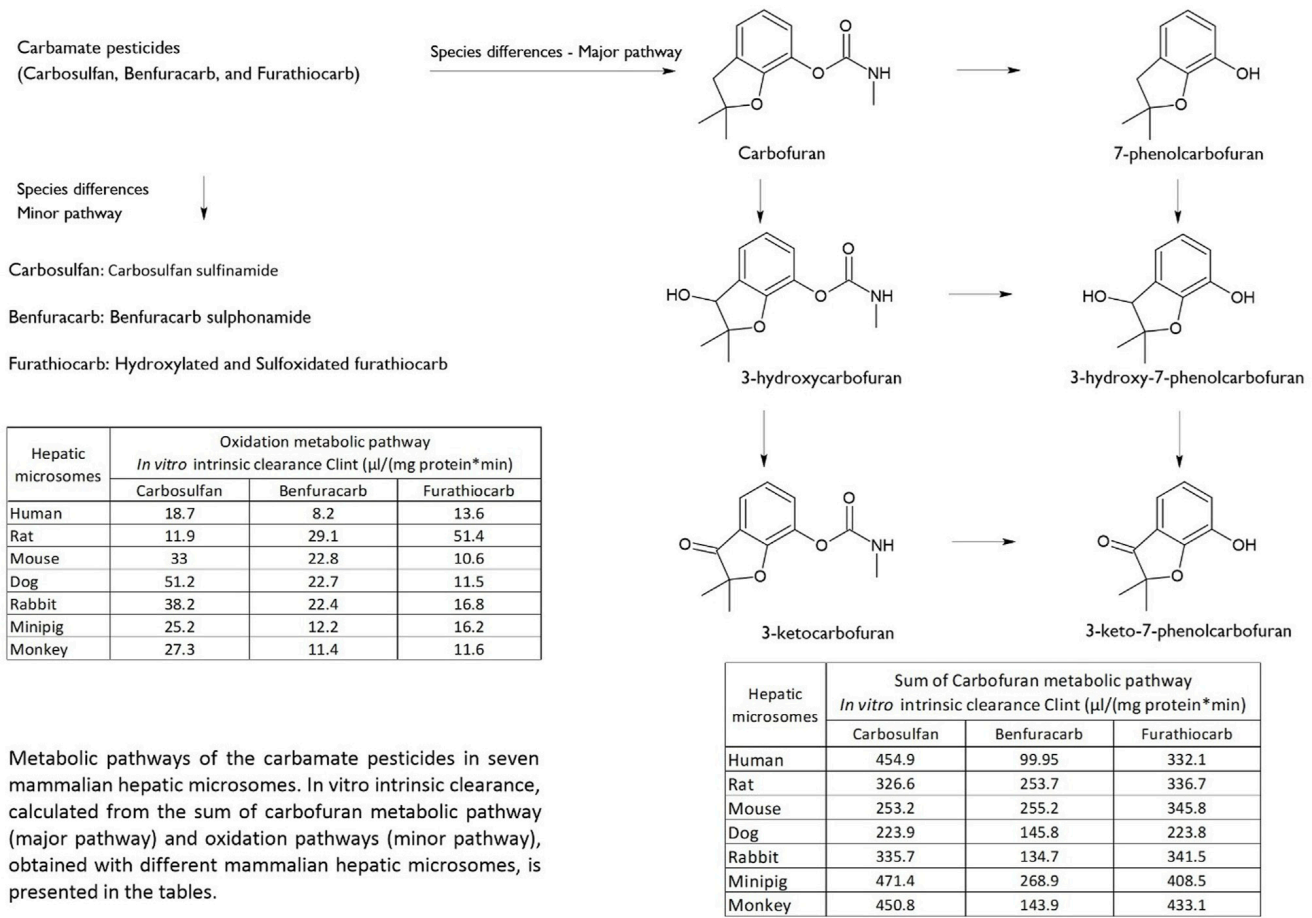
Comparative analysis of carbamate pesticide metabolism across species.

Comprehensive *in vitro* studies on the metabolism of carbamate pesticides, carbosulfan, benfuracarb and furathiocarb, by hepatic microsomes from 7 species including human, rat, mouse, dog, rabbit, minipig, and monkey illustrate the extent of species differences among related carbamates (Figure 1). Utilizing liquid chromatography-mass spectrometry (LC-MS) analysis, a total of eight phase I metabolites were identified. Primary metabolic pathways observed included the oxidation of sulfur and the cleavage of the nitrogen-sulfur bond (N-S) in the parent compounds (Abass et al., 2009; Abass et al., 2014; Abass et al., 2022; Abass et al., 2023). The metabolism of carbamate pesticides to more potent metabolites through the carbofuran pathway is a prevalent feature across all species examined, although there were notable differences in amounts and ratios of the metabolic pathways involved. No unique human metabolites were identified, but there were considerable quantitative differences observed between different species. Whether these differences yield disproportionate human metabolites needing additional studies (see Section ‘Metabolites with a special focus for toxicological assessment’) cannot be decided on the basis of microsomal studies only. These findings highlight the complexity and variability of metabolic pathways across different species and the importance of taking such species differences into account when performing hazard and risk assessment of pesticides.

### Genetic polymorphisms involved in the metabolism of pesticides

Most CYP isoforms as well as other enzymes involved in pesticide’ metabolite(s) formation are genetically polymorphic leading to considerable inter-individual differences in enzymatic activity and kinetics (Ingelman-Sundberg, 1998), internal dose and hence potential susceptibility with regards to the effects of pesticides and their metabolites. The genes encoding for xenobiotic-metabolising enzymes are considered low penetrance susceptibility genes, whose presence confer a low absolute and relative individual risk but may potentially represent a higher population risk. It is therefore the interaction between genes and exposure what makes these genes express their potential role in potential susceptibility to adverse effects. Although mainly genetically determined, the metabolic competence of each organism may also be affected by physiological conditions (developmental stage/age, gender, pregnancy), life style (diet, cigarette smoke, alcohol and drugs consumption), pathological conditions (e,g. hepatic or renal diseases), as well as from exposure to environmental or occupational pollutants, due to induction/inhibition processes. Examples of polymorphic enzymes of relevance to pesticides include several CYP isoforms as well as paraoxonase 1 and several esterases (Darney et al., 2020; Darney et al., 2021; Di Consiglio et al., 2021).

The identification of the isoform-specific metabolism and Michealis-Menten biochemical parameters (Vmax, Km and intrinsic clearance) related to each metabolite by means of specific *in vitro* experimental strategies (see e.g., Timoumi et al., 2019; Santori et al., 2020) can be a good basis to explore human inter-individual differences in kinetics as well as interactions between pesticides (Testai et al., 2021). Indeed, *in vitro* isoform-specific kinetic information can allow improving human risk assessment of single chemicals (and mixtures), describing e potential inter-individual differences and can support the development of robust QIVIVE and PBK models for perform risk assessment in an animal-free environments well as assess interactions among pesticides. Overall, these *in vitro* parameters can be used as inputs for PBK modelling (Testai et al., 2021).

### Metabolic activation

Although metabolism is often described as “inactivation” or “detoxification”, in many cases, metabolites either preserve some activities compared to the parent, or are sometimes bioactivated, becoming - in principle - more toxic or reactive than the parent. Consequent toxicity outcomes depend on the intrinsic activity/reactivity of the metabolites formed, as well on the existence of mechanisms inactivating or mitigating potentially reactive or toxic metabolic intermediates. The activation of phosphorothionates, such as parathion and methyl parathion, serves as a prime example of the involvement of CYP enzymes in the bioactivation of insecticides. These types of insecticides are distinguished by their P=S group, which is activated by CYPs to form their oxon metabolites (P=O), significantly more potent (approximately 1000-fold) than the parent compounds with regards to their anti-cholinesterase properties. This increased potency is also responsible for the acute toxicity of these compounds, targeting the central nervous system (e.g., Halpert et al., 1980; Forsyth and Chambers, 1989). Further, as an example of reactive metabolite production, concomitantly with oxon formation, activated sulphur atoms are released, able to bind irreversibly to the very CYP catalysing the reaction, causing enzyme loss and reduction of the corresponding monooxygenase activity (Halpert et al., 1980).

## Current regulation with a focus on metabolites

### Pesticides and pesticide metabolites exposure to humans and environment

Since the development and application of DDT (dichlorodiphenyltrichloroethane) and the “Silent Spring” book publication (Carson, 1962), pesticides have been under increasing regulation and surveillance. Currently, the principal “tool” to assess potential exposure of humans and the environment to pesticides is the residue definition and consequent enforcement by biomonitoring (EFSA PPR Panel, 2016; EFSA, 2022; OECD, ongoing). Residue definition encompasses pesticide-derived molecules, i.e., metabolites and other transformation products from various biochemical and physicochemical processes, identified in residue trial studies to add to the hazard of pesticide-derived exposures.

### Pesticide metabolites in toxicity testing and regulation

In the pharmaceutical field, requirements regarding metabolism and metabolites have been included in the drug regulation in the EU (EMA), USA (FDA) and elsewhere for decades. The necessity to study metabolites in the context of toxicological assessment was recognised in the early 2000s and the action called MIST (Metabolites In Safety Testing; Smith and Obach, 2006; Schadt et al., 2018) has resulted in recent regulatory guidances. Currently, comparative *in vitro/in vivo* metabolism studies are widely used for pharmaceuticals in various stages of development, from a new chemical entity characterization to a pre-clinical safety assessment (FDA Center for Drug Evaluation and Research, 2020). This knowledge has provided a template to apply analogous experimental designs in the pesticide context. The requirement to perform comparative *in vitro* metabolism studies for pesticide active substances related to human health was set by Commission Regulation (EU) No 283/2013 (EC, 2013) setting out the data requirements for active substances, which establishes in Section 5 “Toxicological and metabolism studies” that *“Comparative in vitro metabolism studies shall be performed on animal species to be used in pivotal studies and on human material (microsomes or intact cell systems) in order to determine the relevance of the toxicological animal data and to guide in the interpretation of findings and in further definition of the testing strategy. An explanation shall be given or further tests shall be carried out where a metabolite is detected in vitro in human material and not in the tested animal species”.* In addition, the same Regulation defines that “*The relevance of generating toxicity data in animal models with dissimilar metabolic profiles to those found in humans shall be addressed, if such metabolic information is available, and taken into consideration for study design and risk assessment”*.

### The *in vivo* toxicokinetic study in rats

**FIGURE 2:**
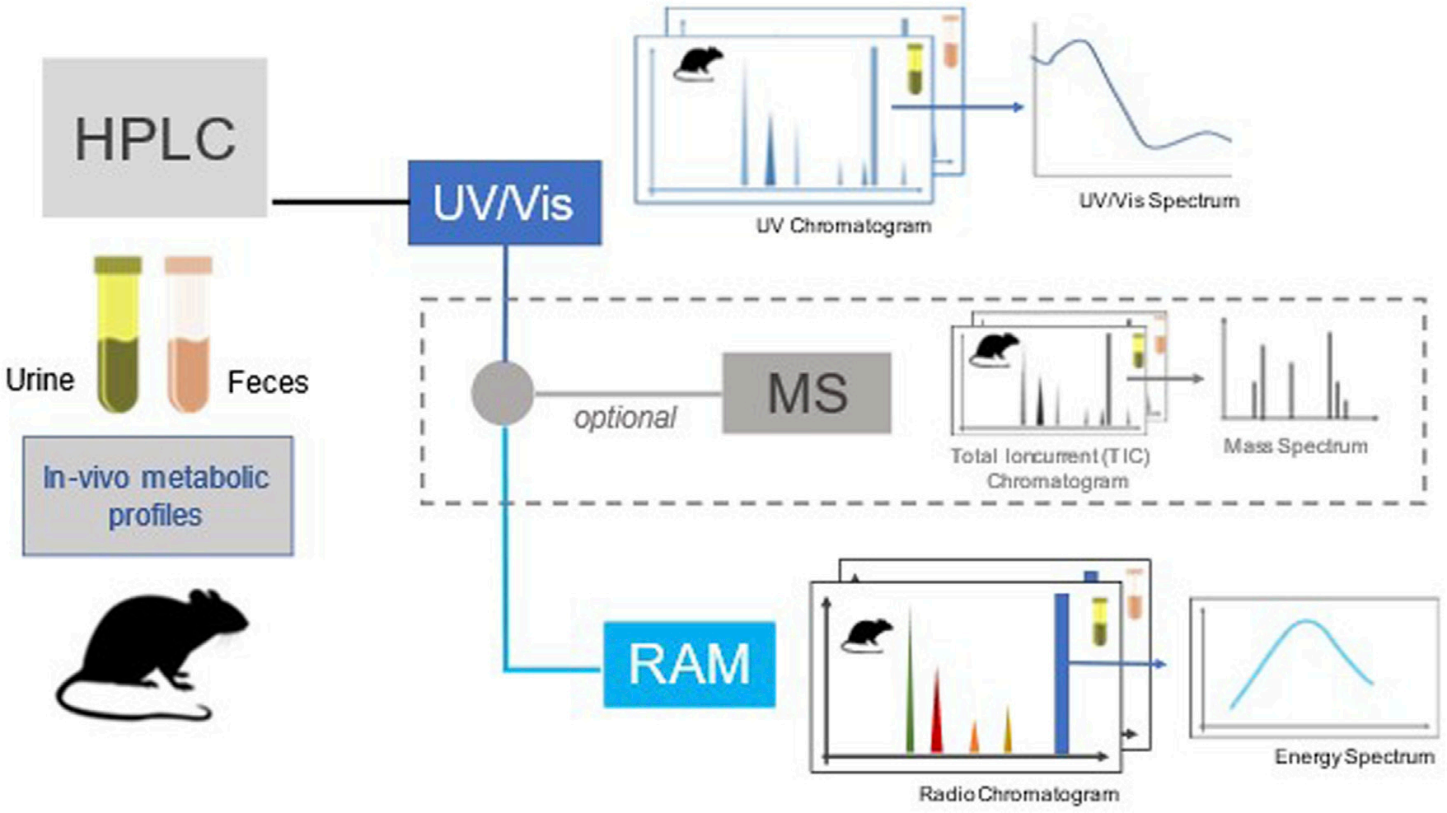
A general outline of the rat *in vivo* toxicokinetic study with analytical modalities for measuring potential metabolites.

Currently, the only regulatory study in which pesticide metabolites should be identified, is the rat *in vivo* toxicokinetic study according to the Organisation for Economic Co-operation and Development Test Guideline 417 (OECD, 2010)^1^. This study should yield information regarding rate and extent of absorption, distribution, excretion, metabolism, metabolite identification and characterisation produced *in vivo* and main results are illustrated with three examples in Table 1. The clearance of the substance and its metabolites, estimated based on radioactivity, in urine, faeces, exhaled air and bile if appropriate, and on the identity of metabolites identified on the basis of radiometric, liquid chromatographic and MS techniques (Figure 2). The principal goals are the measurement of mass balance along different routes of elimination, metabolites profile and their tentative identification, and distribution of radioactivity among different tissues. Toxicokinetic characteristics such as half-life, clearance, etc., can be derived from the study only to an approximate extent and no distinction between parent and metabolites can be made upon quantification of total radioactivity. Nevertheless, a metabolic chart providing the identification of principal metabolites is presented, although quantitative determinations of metabolites are usually reported for only one time point (24 h or longer).

### Comparative *in vitro* metabolism study

**FIGURE 3:**
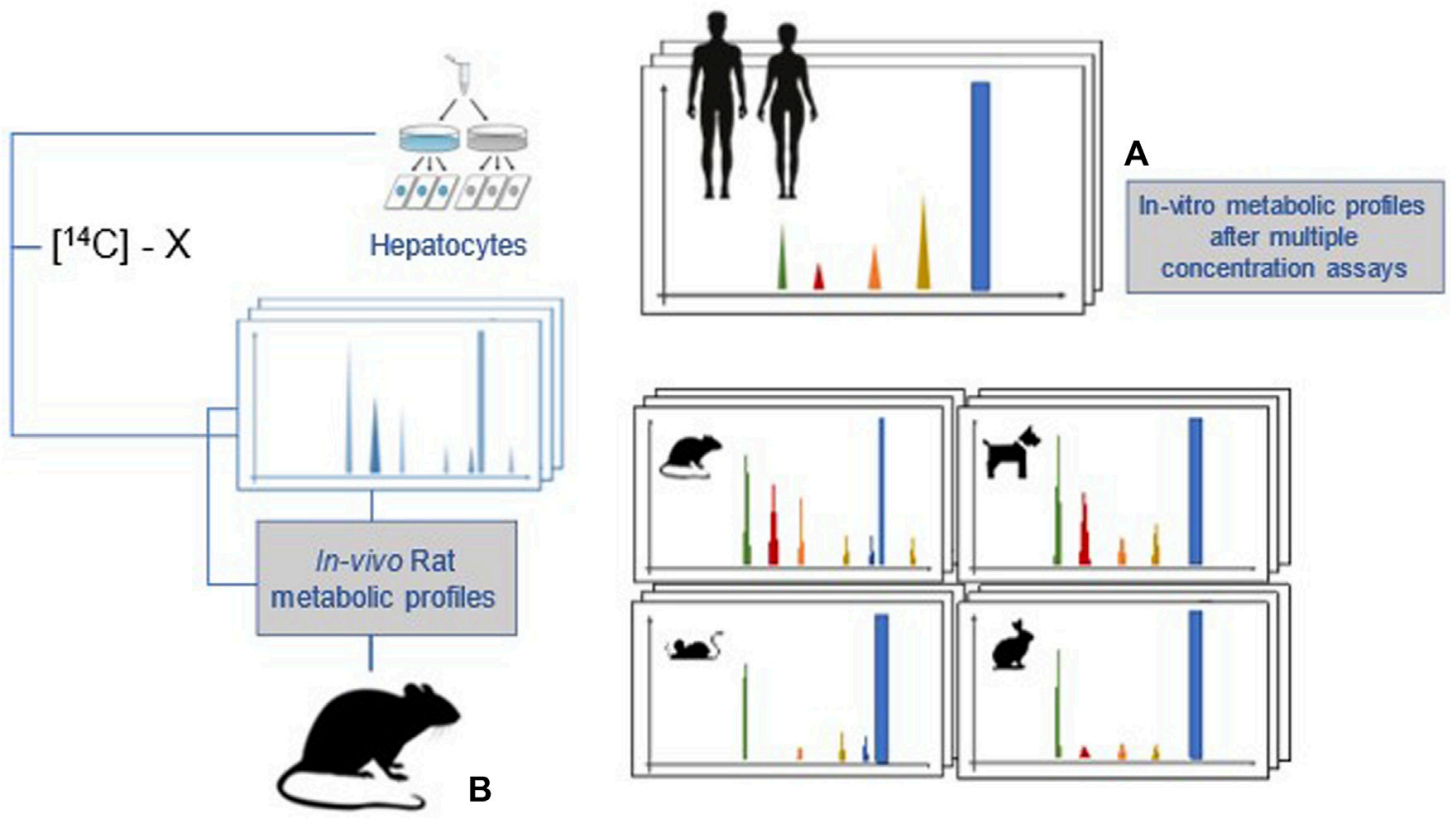
A general outline of the comparative *in vitro* metabolism study (modified from fig in EFSA PPR Panel et al., 2021b). **(A)** Metabolic profiles in hepatocyte from 5 species (human, rat, mouse, dog, rabbit). **(B)** Metabolic profile in rats after in vivo administration.

After 2 years of development, the European Food Safety Authority (EFSA) scientific opinion on comparative *in vitro* metabolism studies was published in 2021 (EFSA PPR Panel et al., 2021b). The main aim of these studies is to evaluate whether all significant metabolites formed in the human *in vitro* test system, as a surrogate of the *in vivo* situation, are also present at comparable level in animal species used in toxicological studies and, therefore, if their potential toxicity has been appropriately covered by animal studies. These studies may also help to decide which animal model, with regard to a specific compound, is the most relevant for human risk assessment (Figure 3). In the experimental setup, primary hepatocytes in suspension or culture are employed as a currently practical and most representative cell system for prediction of *in vivo* metabolites. Because the identification of unique human metabolites (UHM) and disproportionate human metabolites (DHM) is the most important goal of the comparative metabolism study, the experimental design of 3 × 3 × 3 (concentrations, time points, technical replicates, on pooled hepatocytes) will maximise the chance to identify metabolites of possible concern. When DHM and UHM are being assessed, test item-related radioactivity recovery and metabolite profile are the most important parameters. Once detected at concentration >5% of the parent compound, structural characterisation of the assigned metabolites is performed with appropriate analytical techniques. For the toxicological assessment of metabolites, the uncertainty factor approach (only for DHM) is the first alternative to testing option, followed by new approach methodologies (e.g., Quantitative structure-activity relationship (QSAR), read-across, *in vitro* methods); only if these fail, *in vivo* animal toxicity studies are recommended to be performed.

Knowledge of the *in vivo* rat metabolic pathway could be used to compare if all metabolites formed in human hepatocytes are also present in the rat. Depending on the specific chemical, it may be possible that a human metabolite is not present in the rat but in another species. For this reason, it is useful to perform comparative *in vitro* metabolism studies in hepatocytes from all animal species used in toxicity studies (i.e., rat, mouse, dog and rabbit). Although information on metabolic profile is available from the *in vivo* rat toxicokinetic study, rat hepatocytes should also be used to compare rat *in vitro* and *in vivo* metabolites, because difference in these profiles may affect the assessment of potential human disproportionate metabolites. The need for all animal species aims to address the information set in the data requirement of Commission Regulation (EU) No 283/2013 (EC, 2013): “*to determine the relevance of the toxicological animal data and to guide in the interpretation of findings and in further definition of the testing strategy”*.

Elucidation of pesticide metabolism and metabolites in humans and experimental animals is of importance in the context of toxicological risk assessment. It also adds useful pieces of information for detection and evaluation of metabolites in different matrices (e.g., crops, livestock, environment), improves biomonitoring efforts via better toxicokinetic understanding, and ultimately, it develops regulatory schemes employing physiologically based or physiology-mimicking *in silico* and/or *in vitro* test systems to anticipate the exposure of humans to potentially hazardous substances in plant protection products.

### Metabolites of pesticides in the environment, food/water and livestock: Regulatory considerations

After their approved uses, pesticides undergo transformations in various environmental compartments and organisms and these transformation products - not necessarily the same as identified in species used for the toxicity testing - may cause exposure of humans via food, drinking water or environmental pollution. The metabolites that are detected in residue trials and in other environmental studies, are assessed to the extent required by specific regulations.

Regulation (EU) No 283/2013 requires, for dietary exposure assessments, the consideration of the presence of pesticide residues i.e., the active substance and its metabolites, arising from different sources and their aggregate exposure. Pesticide active substances are “data-rich” substances (see EC, 2013). However, this is not necessarily the case for pesticide metabolites found as residues in crops (e.g., apple) and/or animal products (e.g., milk), because there are no specific data requirements for these metabolites (EC, 2013). Since the availability of scientific opinions and recommendations tailored to the assessment of pesticide metabolites in food and feed in Europe, which also aim to reduce unnecessary animal testing (EFSA PPR Panel, 2016), the toxicological assessment of these metabolites has increasingly relied on predictive *in silico* tools such as QSAR, grouping and read-across approaches. The integrated use of these approaches is also recommended in the forthcoming update of the OECD Guidance on the Definition of Residue (ongoing).

The toxicological assessment of residues includes a first tier to assess genotoxicity: residues showing a genotoxic potential will raise a concern that would need risk management consideration in the EU. A second tier, for non-genotoxic residues, may be followed according to their levels in crops and or livestock metabolism studies. The aim of this second tier is to establish whether the toxicological profile of the metabolites is similar or not (e.g., different target organs or critical effects) to the parent compound or another test substance (e.g., structurally similar). In the absence of specific experimental data on the metabolites itself, the assessment of the toxicological profile in this second tier also relies on grouping and read across approaches. Comparing the toxicological profile of the metabolite to the parent would allow grouping (or not) the two compounds for the derivation of the residue definition for risk assessment.

Another source of human exposure is drinking water mainly through surface water and/or groundwater extracted for the production of drinking water. Pesticide transformation products after water treatment will be assessed in the EU following the ECHA/EFSA guidance on water treatment (ECHA/EFSA et al., 2023). The toxicological profile of metabolites of pesticides occurring in groundwater are assessed in the EU following the EC guidance on the relevance of groundwater metabolites (EC, 2003; EC, 2021). For water treatment transformation products, a tiered-approach similar to residue metabolites is followed and it consists of a first tier to assess genotoxicity potential and in the following tiers to set a health-based guidance value for conducting a dietary risk assessment. The trigger for each tier is based on their occurrence in water. The guidance is also promoting the alternatives to animal testing and therefore relied on QSAR, grouping, and read across approaches too. For the groundwater metabolites, as opposed to residue and water treatment transformation products, screening for toxicity is triggered when the parent has toxicological properties of concern, i.e., is classified for reproductive toxicity and/or carcinogenicity. In such cases, it must be shown that the groundwater metabolite does not qualify for the same classification as the parent. Hazard assessment determined based on the Threshold of Toxicological Concern (TTC) concept would trigger a risk assessment, setting of a health-based guidance value (HBGV) would be needed for the metabolite.

## Analytical methods for pesticide metabolites measurement

As already alluded in previous sections, analytical methods are in the heart of metabolite research. Metabolite identification, both *in vivo* and *in vitro*, is routinely performed in the pharmaceutical sector to elucidate metabolic pathways of candidate drugs (Penner et al., 2012). Gathering information regarding the identities, abundances and numbers of metabolites formed is one of the main goals of metabolite research (Spaggiari et al., 2014). Identification of metabolites is also of major importance for detecting structural liabilities of new chemical entities (NCEs) that could affect metabolic stability, elucidating metabolic pathways, predicting potential metabolite toxicity and comparing the metabolite profiles of various species under study with human profiles, so as to select the most appropriate species for *in vivo* toxicity studies. High-throughput *in vitro* metabolism assays combining high pressure liquid chromatography (HPLC) and mass spectrometry (MS) has become the prevailing analytical strategy for *in vitro* metabolism studies (Spaggiari et al., 2014; Tolonen and Pelkonen, 2015). Besides the combination of HPLC and MS, radiolabelled materials are currently routinely used in early drug development and pesticide research and the most common approach is to use ^14^C isotope-labelled compound and the radioactive detector followed by MS/MS according to TG 417 (OECD, 2010). Currently for pesticides, this rat toxicokinetic study is the only guideline toxicokinetic study, but comparative *in vitro* metabolism study has been performed on pesticides (new molecules or renewal procedure) starting from 2013, although a formal guideline has yet to be developed after the adoption of the EFSA Scientific Opinion (EFSA PPR Panel et al., 2021b).

### Current rodent toxicokinetic study guideline for pesticides–analytical considerations

In the pesticides area, according to TG 417, the active substance is radiolabeled and administered to the animals and afterwards all metabolites present at 5% or greater of the administered dose should be identified to provide a metabolic scheme for the test substance. The identification refers to the exact structural determination of relevant metabolites. Some individual examples about general outcomes of this study are presented in Table 1. It is obvious that the number and abundances of metabolites are variable, and comprehensive quantitative analytics is challenging and currently required for ensuring that major metabolites are taken into closer toxicological assessment.

#### Radioactive techniques

The inherently quantitative aspect of radioactivity is crucial for initially establishing toxicokinetic patterns of parent/potential metabolites when structures of metabolites are unknown, and their ultraviolet (UV) spectra and relative MS ionization responses are not known. Therefore, one of the benefits for using radiolabelled compounds is in conjunction with a chromatographic separation method to provide information of the behaviour and amounts of various metabolites as a function of time. Separation of produced metabolites by liquid chromatography is superior to the use of reverse and normal phase thin layer chromatography (TLC), which is currently regarded obsolete.

#### Non-radioactive techniques

There may be cases where non-radioactive material can be sufficient. The questions about the use of high-resolution mass spectrometry (HRMS) methods to circumvent the need for radioactive material in identifying metabolites in complex mixtures is controversial and considering the examples provided (Guo et al., 2020), it appears that radioactive and non-radioactive approaches are complementary in this context (Kaufmann, 2020). However, non-radioactive substances could be used for metabolism studies if certain prerequisites are considered: although metabolite quantitation and recovery are more difficult to measure with non-labelled compounds, comparative assay conditions–to the extent it is possible–should ascertain, that individual metabolites could be compared across target matrices, be they of *in vitro*, *in vivo*, environmental, or other origins or conditions. Absolute recoveries are more difficult to measure, but this uncertainty is probably not too large to prevent metabolite comparisons.

#### Analysis of stereoisomers

Most chiral compounds exhibit only slight differences in metabolism; however, some notable examples are available where rat *in vivo* studies show major differences in metabolism of enantiomers and consequently it is important to identify whether interspecies differences in enzyme activity can alter stereospecificity of enzymes (UK DEFRA, 2003). *In vitro* metabolism studies are useful to address the potential stereo-selective metabolism of chiral pesticide active substances, and therefore, the methods of analysis to be used for *in vitro* metabolism should be stereo-specific (EFSA, 2019).

If enantioselective metabolism has been observed in early *in vitro* or *in vivo* studies, further inquiries of potential enantioselective toxicity should be consequently planned and assessed in regulatory toxicity studies (see Ji et al., 2023), preferably beginning with *in silico*/*in vitro* toxicity studies (see section Investigating toxicity of pesticide metabolites).

### Comparative *in vitro* metabolism study—importance of analytical methods

The study design of an *in vitro* comparative metabolism study, described in some detail in earlier section, might influence the detection of relevant metabolites. There are many experimental and technical conditions that can impact on metabolite detection and identification. For instance, the concentration tested *in vitro* may be too low and consequently miss metabolites at low levels. The number of cells used, and duration of the incubation time could influence the amounts of metabolites formed. Overall, although some metabolites might evade detection owing to lack of ionisation or large differences in structure and/or MS fragmentation patterns versus the parent compound (rendering MS data unrecognizable compared with the parent compound), examining plasma samples for metabolites has been a relatively reliable method (Dahmane et al., 2014; Kirchmair et al., 2015).

Because the metabolic profile of a new pesticide in human and other species is unknown *a priori*, the metabolite identification should be performed by using dedicated analytical approaches, including various modes of data acquisition. In the context of *in vitro* comparative metabolism studies, untargeted assays involve comparing the human hepatocytes and animals’ derived hepatocytes to identify potential differences between their metabolite profiles. The sensitivity of the analytical methodology employed when performing untargeted assays could strongly influence the detection of relevant metabolites. Hence, some metabolites formed might be lower than the limit of detection (LOD) of the analytical method and/or in relatively low concentration level compared to the parent compound.

### Biological/toxicological/regulatory significance of analytical methods

It is perhaps a self-evident statement that the most modern LC–MS techniques, which make use of ultrahigh chromatographic performance and high-resolution mass separation, are the best tools for the identification and quantification of pesticides and their metabolites particularly for assessing their *in vitro* and *in vivo* toxicokinetics and their relationships with toxicological risk assessment, at least for most small molecular organic substances (Tolonen and Pelkonen, 2015). However, it must be noted, that a single tool cannot completely cover the huge variability of pesticide chemical space, and consequently several techniques for separation and for detection, identification and quantification are needed. Furthermore, in all circumstances, experimental design conditions as well as proper sample handling techniques are crucial for creating the necessary preconditions to acquire toxicologically meaningful analytical results (Pelkonen et al., 2013). Finally, the reliability and robustness of quantification of a pesticide and its metabolites as well as the expected biological significance of the concentrations of the parent/metabolites measured are heavily dependent on test system-specific experimental and biological factors such as binding to proteins or surfaces (“free fraction”), stability in the system, and many other considerations (Pelkonen et al., 2009a; Heuberger et al., 2013). Only after the thorough consideration of all the possible factors in the *in vitro* or *in vivo* system itself, it is possible for the analytical technique to produce a result that is conducive to reliable toxicokinetic and toxicodynamic assessment and that ultimately contributes, within its own limitations, to the most reliable regulatory decision.

## Experimental test systems to study metabolism and metabolising enzymes

In contrast to pharmaceuticals, human *in vivo* studies with pesticide active substances are limitedly performed. Few examples are available on orally/dermally controlled exposure studies with measurement of the active substance and (selected) metabolites in urine (Harada et al., 2016; Faniband et al., 2019; Oerlemans et al., 2019; Wrobel et al., 2022). In general, these toxicokinetic (TK) studies with pesticide active substances provide limited information compared to pharmacokinetic (PK) studies with pharmaceuticals, i.e., only time-dependent information on urinary levels of parent and selected metabolites have been reported, whereas information on internal dosimetry is lacking. Thus, for pesticide active substances, possibilities to have information on metabolism and metabolites depend mainly on animal *in vivo* and *in vitro* studies and human *in vitro* studies. As explained above, an *in vivo* toxicokinetic study in rats, in line with TG 417 (OECD, 2010), is the principal regulatory study to yield information about clearance of the substance and its metabolites (estimated based on radioactivity) from the rat with excretion into urine, faeces and exhaled air. Information on the structure of metabolites (ideally identified based on MS techniques and confirmed with standards) is typically provided for excretion into urine, faeces and sometimes for bile. Based on such a study, insight into the fate of the compound regarding metabolism and routes of elimination (metabolism, excretion, exhalation, etc.), and about the (tentative) structures of principal metabolites can be obtained. Similar *in vivo* toxicokinetic studies are not required in other species used for toxicity testing, and such data is therefore typically only available for rats. Consequently, the best remaining and practically available way to obtain metabolite information in other species is to use *in vitro* techniques.

### Prerequisites for the selection of test systems

There are three principal prerequisites for the selection of a proper *in vitro* test system to compare metabolism of pesticides between different species (EFSA PPR Panel et al., 2021b). The first one is that the system recapitulates the most important *in vivo* metabolizing organ, i.e., liver, so should be derived from liver or mimic liver in terms of xenobiotic metabolism function. It should be stressed that no single *in vitro* test system can mimic all the complexities of human (or animal) TK processes, but from the metabolism point of view, the liver is considered the most important organ for most pesticides that are metabolized to a significant extent. The second prerequisite is that the test system selected should preferably have been validated. If not validated, there should preferably be a history of use with related best practices for metabolism studies, so that researchers and regulators have sufficient confidence in its performance (not necessarily in the pesticide area). In any situation, functional characterization of the test system (i.e., assessment of enzyme activities based on conversion of probe substrates, e.g., as described by Tolonen et al. (2007)) should be performed in the laboratory that performs the metabolism studies. The third prerequisite is to have workable liver-derived test systems from the species of pivotal toxicity tests.

The most used *in vitro* hepatic test systems for the formation, identification and quantitation of metabolites are listed and described in Table 2 (and references therein). Hepatic test systems span from individual enzymes and subcellular preparations to isolated hepatocytes and liver slices (e.g., Pelkonen et al., 2009b; Buratti et al., 2011; Buratti et al., 2013; Lake and Price, 2013; Buratti and Testai, 2015; Wilk-Zasadna et al., 2015; Gouliarmou et al., 2018; Bernasconi et al., 2019; Gao et al., 2020). Current research has been focused on developing various experimental setups (matrices, spheroids), inexhaustible sources for functional cells (embryonic stem cells, pluripotent stem cells) and more complex systems, including induced self-organized tissues (organoids) and the use of microfluidic technologies (organ-on-a-chip) (e.g., Wilk-Zasadna et al., 2015; Bell et al., 2017; Burton et al., 2018; Gouliarmou et al., 2018; Gough et al., 2021; Rusyn and Roth, 2021; Rusyn et al., 2022).

**TABLE 2:** Commonly used in vitro hepatic test systems used for the production, identification and quantitation of metabolites: advantages and disadvantages. Modified from Wilk-Zasadna et al. (2015) and Gouliarmou et al. (2018).

Test system	Advantages	Disadvantages	References (examples)
Purified enzymes, Recombinant enzymes	• identification of an intrinsic metabolism of a pesticide	lack of genuine environment	Buratti et al. (2011)
Liver microsomes and subcellular fractions	• major phase I enzymes present (CYPs etc.)	• lack of important enzymes and natural environment	Buratti et al. (2013), Buratti and Testai. (2015)
	• individual variation		
Liver S9 or homogenate	• major phase I and II enzymes present	• cellular and organ architecture lost	Pelkonen et al. (2009b)
	• metabolic profiles and (sub)clearance estimations	• multiple cofactors required	
Primary hepatocytes, in suspension or plated	functionally active whole cells	• viable for a limited time in suspension; gradual decline of metabolic functions in time	Bernasconi et al. (2019)
HepaRG^®^ cell line	• metabolic capacities comparable to primary human hepatocytes	• represents one donor only	validated by EURL-ECVAM for CYP induction Bernasconi et al. (2019)
Liver slices	• basic hepatic architecture preserved	• specialized preparation and incubation techniques	Lake and Price (2013)
		• difficult to obtain	
Hepatocyte-like cells based on pluripotent (embryonic or induced) stem cells	• Easy access to material from different donors (for induced pluripotent stem cells)	• limited information on performance available	Gao et al. (2020)
		• functionality in doubt	
Advanced test systems based on isolated hepatocytes and/or supporting cells (examples)			
Currently investigated, but not yet established, test systems for assessing human liver metabolism (see Wilk-Zasadna et al. (2015); Lauschke et al. (2016); Serras et al. (2021)			
Primary cell co-cultures	• possibly physiologically relevant interactions with other cell types	• limited information on performance available	Guguen-Guillouzo and Guillouzo (2010)
			Chan et al. (2013)
Microphysiological systems with microfluidic flow, e.g., HµREL^®^ Biochip	• improved stability and functionality	• limited information on performance available relative complex setup	Burton et al. (2018)
	• commercially available		Rusyn and Roth 2021; Rusyn et al., 2022
Spheroid scaffold-free cultures	• improved stability and functionality	• limited information on performance available	Bell et al. (2017)
	• relatively simple setup		
Organoids based on primary hepatic material	• close resemblance to *in vivo* physiological situation	• limited information on performance available	Gough et al. (2021)

### Hepatocytes

Isolated primary hepatocytes, either suspended (for short-term) or plated (for longer-term), fulfill many of the abovementioned prerequisites. Hepatocytes represent intact liver in terms of cellular organization and function, contain the most important biotransformation enzymes and there are decades of experience in using them in research and drug development (Sevior et al., 2012). They are also commercially available. Although human hepatocytes have not been validated as a test system for comparative metabolism studies, it is of interest to note that they have been validated regarding CYP induction (Bernasconi et al., 2019). Given that decades of experience exist in using them for elucidating metabolism and clearance in drug development by pharmaceutical industry, they are considered an appropriate test system for comparative metabolism studies. Hepatocytes isolated from different animal species, including rat, mouse, dog and rabbit have been used in research and drug development for a long time (e.g., Chesné et al., 1993; Tuschl et al., 2008). The hepatocyte test system from a fish species, rainbow trout, for the determination of *in vitro* intrinsic clearance has been developed in connection with OECD, for which a technical guideline is available (OECD, 2018). It should be noted that human liver slices, which can be considered as a test system closer to the *in vivo* situation, although extensively applied in basic research (Lake and Price, 2013), is not a practical test system for the purposes of *in vitro* comparative metabolism studies, as access to standardized well-characterized material from different species would be a challenge.

Permanent hepatocyte cell lines originating from human (or animal) liver are used for research and screening, but most of them lack sufficient levels of biotransformation enzymes. The HepaRG^®^ cell line, which has been validated as CYP induction test system (Bernasconi et al., 2019), is an exception since it contains most biotransformation activities at sufficient levels, shown to be quantitatively comparable to human hepatocytes for prediction of clearance of several cytochrome P450 substrates (e.g., Zanelli et al., 2012). It must be noted, though, that its origin from a single individual raises concerns about its representability for human metabolism. However, human population variability can be extrapolated from *in vitro* testing using PBK modelling that includes intra-species variability.

### Liver tissue fractions

Various hepatic tissue fractions (e.g., microsomes or cytosol) have been used for decades in experimental research and also for regulatory purposes, and they have contributed to the advancement of toxicokinetic research (e.g., Pelkonen et al., 2009a; Buratti et al., 2013; Buratti and Testai, 2015). However, since subcellular fractions are not endowed with the complete drug metabolising enzymes, there is at present no advantage in using them in the *in vitro* comparative metabolism study, except perhaps as a screening tool or for providing additional evidence for the role of microsomal, mitochondrial and/or cytoplasmic enzymes in the isoform-specific metabolism of the compound under study, in combination with the use of single recombinant enzymes.

### Use of extrahepatic tissues

As indicated above, from the metabolism point of view, the liver is considered the most important organ for most pesticides that are metabolized to a significant extent. When *in vivo* produced rat metabolites point to predominantly products of CYP reactions (hydroxylations, dealkylations, etc.) or conjugation reactions with glucuronide (by UDP-glucuronosyltransferases (UGTs)), sulfate (by Sulfotransferases (SULTs)), glutathione (by Glutathione S-transferases (GSTs)) or acetyl (by N-acetyltransferases (NATs)), a liver-based tool is considered sufficient to use (EFSA PPR Panel et al., 2021b). In specific cases, the Panel suggests to consider the use of a non-liver-based test system, in which metabolism takes place, for a better understanding of species differences. This may especially be relevant if the parent chemical is rapidly converted by a non-liver tissue (e.g., intestine in case of oral exposure), before reaching the liver, resulting in limited liver exposure to the parent chemical. This may be the case when *in vivo* (rat) metabolites are predominantly products of carboxylesterases, which catalyze hydrolysis of ester bonds. The EFSA PPR Panel Opinion on comparative *in vitro* metabolism studies indicates that if, based on the *in vivo* rat toxicokinetic study, the conversion rate is high (i.e., practically all the parent seems to be initially hydrolyzed), one should consider using both a liver-based tool and a suitable extrahepatic tool relevant for the exposure route, i.e., an enterocyte test system for oral exposure or blood for other routes. If hydrolysis is very rapid in the enterocyte test system, systemic exposure is expected to be mainly to the principal product(s) of hydrolysis, and the principal hydrolysed metabolite should be used as a substrate for the liver-based test system to evaluate its further phase I and II metabolic fates (EFSA PPR Panel et al., 2021b). The enterocyte testing system (or analogous) could be used to evaluate the extent of first-pass metabolism and the extent of exposure of the liver to the predominant hydrolysis product (e.g., Arian et al., 2022).

Regarding CYP activity in extrahepatic tissues, although the gut is more restricted in its biotransformation capacity in terms of enzymes and their activities, its contribution as the first-pass site may be significant, e.g., for CYP3A4/5 substrates (Kato, 2008). Other extrahepatic tissues are assumed to be of little significance in contributing to the *in vivo* metabolite profile, since it has not been reported that specific metabolites are formed in non-hepatic tissues (Gundert-Remy et al., 2014). However, non-liver metabolism and tools to measure it must be considered on a case-by-case manner (EFSA PPR Panel et al., 2021b).

### Considerations on *in vivo*/*in vitro*/*in silico* studies

As discussed above, *in vivo* studies in humans on pesticides are not possible to conduct for ethical reason, at least for regulatory purposes, and also animal *in vivo* studies within regulatory requirements are becoming under increased scrutiny, and, in any case, even those that are currently performed for studying various adversities, generally do not involve metabolic or kinetic measurements. Therefore, the practical goal regarding comparative *in vitro* metabolism studies (EFSA PPR Panel et al., 2021b) is to determine if the *in vitro* metabolic profile found in human hepatocytes would be similar or not to the one obtained when using hepatocytes from the animal species that are used for pivotal toxicity studies (i.e., rat, mouse, dog and rabbit). The comparison of *in vitro* metabolism is focused on evaluating if all metabolites formed in human hepatocytes are also present, and thus also evidenced, in one or more of the animal species used in toxicological studies.

*In silico* prediction of metabolism and potentially also metabolites of concern (see the next section) would be an important goal. Currently used approaches such as QSAR systems are considered not yet sufficiently reliable but would provide additional useful information for regulatory work. Especially in the drug development field, *in silico* tools are being extensively used and could provide useful examples for pesticides toxicokinetics work (e.g., Fagerholm, 2007; Najjar et al., 2023; Rai et al., 2023), but their validity to predict metabolism data in a regulatory setting is so far considered limited.

## Metabolites with a special focus for toxicological assessment

### Comparative *in vitro* metabolism of pesticides

The biological relevance of the test species for toxicity testing and human risk assessment has been ongoing for many decades. Historically, toxicodynamic aspects were the focus and potential interspecies differences in metabolism and resulting metabolites has only been addressed recently in the pesticide regulation (see Section Current regulation with a focus on metabolites). As indicated, no regulatory binding guidance neither any OECD guidelines to be used by the pesticide industry has been produced yet, but recently the EFSA Scientific Opinion on comparative *in vitro* metabolism has been published (EFSA PPR Panel et al., 2021b). In the context of this scientific opinion, the comparative *in vitro* metabolism of pesticides is used as a detailed example to investigate the production of unique and disproportionate human metabolites compared to test species and therefore to characterise the biological relevance of test species for toxicity testing. It is noted that this detailed case example does not correspond to the assessment of other metabolites of concern described within the EU/OECD framework (such as relevant metabolites).

### Unique and disproportionate human metabolites

It is a commonly held opinion that metabolites are implicitly tested in *in vivo* toxicological studies using the parent compound since they are produced *in vivo* in experimental animals during the testing period and any adverse effects, if elicited by them, would be observed. However, qualitative or quantitative differences in the profile of metabolites across species may be observed. Qualitative differences can originate, because the relevant metabolising enzyme may be lacking or may show differences in a particular test species used for toxicity testing. If a metabolite is formed in a mammalian species used for toxicity testing and is not formed in humans, this will in most of the cases, have little for the risk assessment with the exceptional case that the metabolite formed in the animal is extremely toxic. However, this may lead to conservative evaluations for a human health perspective, in which case, the hazard characterisation would indicate high toxicity resulting in low acceptable daily intakes (ADIs) and conservative estimates for humans. In contrast, if a metabolite, formed *in vitro* in humans or the test species or *in vivo* is not formed in an *in vivo* or in an *in vitro* animal test system, this will result in a potential situation of concern, because of potential undetected toxicity of this metabolite, since not tested. Therefore, this situation may need further toxicity testing of this metabolite. This situation is described by the term “unique human metabolite” (UHM), which is defined as a metabolite detected only in the human-derived test system and not in one of the *in vitro* animal-derived test systems or in an *in vivo* study in a laboratory species.

Quantitative differences can be observed due to various reasons, like lower expression or different kinetics of the enzyme, a different (iso)enzyme catalysing the metabolite production, or a different rate of further metabolism of the metabolite (Thompson et al., 2011). Large species differences in quantitative proportions of metabolites may be present even if the metabolite profiles are qualitatively similar (e.g., Pelkonen et al., 2009b; Thompson et al., 2016). For quantitative interspecies differences, two scenarios can be differentiated. First, the quantity of a metabolite may be higher in animals compared to that in humans. In this case, the risk assessment of a pesticide is driven through hazard identification and hazard characterisation of the metabolite. In the second scenario, the quantity of a metabolite may be higher in humans compared to that in animals, with the consequences that effects seen in animals in toxicity testing may not be representatives of effects in humans. In this case, the term “disproportionate human metabolite” (DHM) has been coined and defined as a metabolite that is present at a quantity higher than 4 times in humans when compared to laboratory animal species at any sampling point. The factor of 4 is derived from the toxicokinetic interspecies subfactor of the default uncertainty factor of 100 as defined by International Programme on Chemical Safety (IPCS, 2005). The factor 4 is theoretically accounting for the differences between animal and humans in TK which are mainly due to differences in metabolism.

In some cases, the toxicological assessment of human metabolites, identified as UHM and/or DHM, could make use of already produced data, because these metabolites might have been identified in residues and livestock or groundwater and might have been already tested to assess their relevance for human health. If such data are not available, a possible approach to address the toxicity of a DHM (but obviously not UHM) could be the larger “uncertainty factor (UF)” approach, which is applied with the aim to avoid testing of the metabolite in additional animal studies (see an example in Box 2).

If testing results are not available concerning UHM and DHM or the larger UF approach cannot be applied, the toxicity of the metabolite has to be characterised. In a first step, alternative approaches to animal testing should be employed such as read-across, *in silico* prediction tools and/or *in vitro* methods. Further discussion of testing options is presented in the section ‘Investigating the toxicity of pesticide metabolites’.

### Reactive metabolites

Reactive metabolites, formed by metabolism in the body, are electrophilic species which can react with macromolecules, such as proteins or DNA, by covalent binding. Modified macromolecules may affect cellular functions and lead to various toxic effects (Pessayre, 1993; Pelkonen et al., 2015).

The formation of reactive metabolites can be mediated by different drug metabolizing enzymes, mostly by CYP enzymes. The classic example of the formation of a reactive metabolite is acetaminophen when the administered dose overwhelms the detoxication pathway. Acetaminophen is metabolised in the liver by CYP2E1, CYP1A2, CYP3A4 to a quinone imine reactive metabolite, which in a further step is conjugated with glutathione. The conjugation product is excreted in the urine. With overdoses of acetaminophen the concentration of the quinone imine reactive metabolite exceeds the intracellular glutathione capacity resulting in cellular glutathione depletion followed by oxidative stress, covalent binding to hepatic proteins, and hepatocellular necrosis (Hinson et al., 2010). Further examples for liver toxicity, discussed in detail in the review of Park et al. (2005) are tamoxifen, diclofenac, and troglitazone. Due to risk of hepatotoxicity troglitazone was withdrawn in 2000 by FDA. Mechanistic explanation was provided by Smith (2003) demonstrating the role of the metabolite of rosiglitazone. In addition, impaired bile acid transport due to the parent drug, however also by its sulfate metabolite, was demonstrated (Marion et al., 2007). A recent molecular protein docking study showed targets of troglitazone which were 3-oxo-5-beta-steroid 4-dehydrogenase, neutrophil collagenase, stromelysin-1, and VLCAD and could add to the explanation of troglitazones’ hepatoxicity (Kores et al., 2021).

Other examples of reactive intermediates include alkenylbenzenes, which are natural constituents of a range of herbs and spices and these are bioactivated via phase I reactions (CYP-mediated), resulting in 1′hydroxyalkenylbenzenes and subsequent conjugation via phase II reactions (SULT-mediated), resulting in electrophilic 1’-sulfooxy derivatives which bind (amongst others) to DNA and are considered responsible for their genotoxicity and carcinogenicity (Rietjens et al., 2005). Further well-known examples are for the metabolic activation and the resulting genotoxicity and carcinogenicity are aflatoxin B1 (Shimada and Guengerich, 1989) and aromatic and heterocyclic amines (Yueh et al., 2001).

There are also data suggesting that reactive/cytotoxic metabolites could be involved in the development of hypersensitivity reactions. One prominent example is the inhalation anesthetic halothane, now retracted from the market because of causing severe liver injury. There is convincing evidence that its acid chloride metabolite, a minor metabolite, is the culprit for this adverse reaction and the mechanism was thought to be immune related (Farrell, 1988). Two hypothetical mechanisms are discussed. Firstly, reactive metabolites bind to proteins. These proteins are recognized as antigen by antigen-presenting cells which elicits a cascade of immune responses. A second mechanism may be deduced by the property of inducing apoptosis, necrosis and finally cell death, with following steps of releasing intracellular molecules (until now not identified) so-called “danger signals”, which induce in the immune cells the immune reaction (reviewed by Elzagallaai and Rieder, 2022).

In order to evaluate the potential toxicity of a reactive metabolite in humans, several authors have proposed to study the covalent binding in a quantitative manner in relevant cells, e.g., liver cells in a concentration dependent manner (Nakayama et al., 2009) and to compare the concentration response findings with estimated exposure. Further proposals consist of a battery of tests assessing toxicity by assays that quantify CYP-dependent and/or CYP-independent cell toxicity, mitochondrial impairment and inhibition of the bile salt export pump (Thompson et al., 2011; Thompson et al., 2016). However, studies aiming to identify the presence of reactive metabolites are presently not a regulatory requirement. Because reactive metabolites may escape the identification due to their short presence in the *in vitro* assay, it should be considered to introduce the techniques available to identify them in order to identify early on the potential for hepatic injury. Zeller et al. (2020) have presented interesting case studies illustrating how to assess the genotoxic potential of human metabolites whereby their special focus was on the compound class of aromatic amines, potentially genotoxic carcinogens, because of its use as building blocks of pharmaceuticals. The same group has described in their review (Schadt et al., 2018) that following the metabolites in safety testing (MIST) regulatory guidance (FDA, 2008) has influenced industries’ approaches identifying drug metabolites and their potential contribution to safety, focusing on comparative metabolic profiles, human versus test animals, with specific emphasis on the metabolic profile in plasma. Their examples show that this latter information provides a fuller picture. However, within the pesticide area human experimentation is restricted, thus metabolite comparison in the blood of exposed humans and animals would be rarely feasible.

BOX 2 **Disproportionate Human Metabolite (DHM): how to calculate an additional uncertainty factor.**

A possible theoretical approach to address the toxicity of a DHM (but obviously not UHM) could be the larger “uncertainty factor” approach, aiming to avoid testing of the metabolite in additional animal studies. When extrapolating from the reference point (RF) obtained from the results of animal studies, an adjustment factor is applied to account for the interspecies and the intra-human variability to derive the ADI. The DHM has been co-tested in animal studies. However, because of the lower production in the animal the tested amounts are – after applying the modified UF of 25 for the metabolite in which the UF of 4 for interspecies difference in toxicokinetics has been excluded – lower than the exposure to this metabolite in humans at the ADI. Assuming that the DHM is the toxic moiety, a conservative option is to apply an additional uncertainty factor to the conventional default factor to adjust the levels of the metabolite tested in animals and those present in humans at the ADI. This approach would result in an overall larger UF for deriving a health-based guidance value for the parent compound. An appropriate overall UF can be calculated which is higher than the default value of 100 depending on the relative quantity of the DHM compared to animal species. For example, we assume that the quantity of the disproportionate metabolite in rat is 5% and the quantity in humans is 30% (6-fold higher) of the dose. In this example, assuming a reference point of 100 mg/kg body weight (bw) per day for the parent compound, from 100 mg/kg bw the resulting metabolite is 5 mg/kg bw per day (=5%) which has been tested in the study from which the reference point has been obtained. The ADI, based on the parent compound, would be 1 mg/kg bw per day applying the default uncertainty factor of 100. The safe level of the metabolite in humans would result in 0.2 mg/kg bw per day applying the modified uncertainty factor of 25. In humans, at the ADI, the internal exposure for the metabolite is 0.3 mg/kg bw per day which is higher than the safe level of the metabolite of 0.2 mg/kg bw per day. Applying an additional UF, in this example an additional UF of 1.5 resulting in an overall UF of 150, the ADI for the parent compound would be 0.67 mg/kg bw per day. At the ADI of 0.67 mg/kg bw per day the internal human exposure to the metabolite would be 30% of 0.67 mg/kg per day, equal to 0.2 mg/kg bw per day. Lowering the HBGV as described would adequately protect humans to the toxicity of the parent compound and the disproportionate metabolite.

## Investigating the toxicity of pesticide metabolites

Currently, for pesticides a full toxicological data package is generally available. However, for many metabolites of pesticides potentially exposing humans, empirical toxicity data are often lacking. It would be possible to assess the potential toxicity of these substances in animal studies and it is still mandated to a significant extent by regulations, especially regarding those metabolites included in the residue definition (see Introduction). However, such studies are undesirable in view of the world-wide effort to reduce animal testing for ethical reasons, and in addition such studies are time consuming and expensive. Therefore, non-animal testing methods, also referred to as new approach methodologies (NAM) are the first choice to obtain information on the potential toxicity of identified metabolites under study, as is recognized by major international regulatory bodies. For instance, EFSA has prioritised NAMs as requiring strategic considerations regarding the future of scientific assessments, and a roadmap for action was published (EFSA et al., 2022; Escher et al., 2022; Cattaneo et al., 2023). US-EPA is evaluating and applying NAMs for assessing risks to human health and US-EPA’s Office of Research and Development is actively developing, testing, and applying NAMs (US-EPA, 2018; US-EPA, 2021; US-EPA, 2023). The FAO/WHO joint meeting on pesticide residues encourages sponsors to submit data generated using NAMs (FAO/WHO, 2018). In 2018, the Interagency Coordinating Committee on the Validation of Alternative Methods (ICCVAM) of the National Toxicology Program (NTP) of the USA published a roadmap on the development of NAMs (ICCVAM, 2018).

### In silico approaches

As the first step, it may be investigated whether data on genotoxicity or general toxicity exist for structurally similar substances. This data can then be used to tentatively predict toxic properties of a metabolite. Such information may come from QSAR models and read across to chemicals with similar structures (EFSA PPR Panel, 2016; Benigni et al., 2019). QSAR models are statistical-based or knowledge-based models that link chemical structure and toxicological activity in a quantitative manner for several compounds. Read across approach is a technique used for predicting endpoint information for one substance by using data from the same endpoint from one or more other substances which are structurally related. Both QSAR and read across are currently able to provide qualitative or at best semiquantitative data on toxic potential and have extensively used and accepted also in the regulatory arena.

The application of QSAR and read across for genotoxicity and general toxicity, and some worked examples, have been previously described in detail in the PPR Panel Guidance on the establishment of the residue definition for dietary risk assessment (EFSA PPR Panel, 2016). Further guidance on grouping and read across has been published by ECHA (2008, 2013), OECD (2010) and EFSA (2019) and practical information as well as examples are available in agencies’ home pages.

QSARs are mostly used for predicting genotoxic properties of substances with a known chemical structure due to the availability of several tools giving rise to good predictions, while their use in predicting general toxicity is limited (Madden et al., 2020). When using QSARs, conclusions on the genotoxicity should be drawn on the outcome of two or more independent QSAR models for each genotoxicity endpoint (Worth et al., 2010; Worth et al., 2011; ICH, 2014; EFSA PPR Panel, 2016). All relevant genotoxicity endpoints, i.e., gene mutations, and structural and numerical chromosomal aberrations have to be considered. The conclusion on the genotoxic potential of the metabolite/s should be justified by providing all necessary information, e.g., which models were used, the applicability domain and reliability of the models, etc.

### *In vitro* approaches

Genotoxicity testing of pesticide metabolites (if needed) has been outlined in EFSA’s genotoxicity testing strategies (EFSA PPR Panel, 2016). It is important to consider the structure of the metabolite because this could affect the behaviour of the compound, e.g., in the penetration to the test cell depending on the change of lipid solubility or in being a ligand for a transporter. Also, the introduction of the metabolizing system needs consideration, because it is possible that a metabolite is further metabolized, possibly into a reactive intermediate. Various *in vitro* genotoxicity tests have been included in the OECD guideline programme (OECD Test Guidelines for Chemicals, see https://www.oecd.org/chemicalsafety/testing/oecdguidelinesforthetestingofchemicals.htm).

Alternative test methods and strategies (NAMs as the foremost example) to measure various toxicokinetic processes (intestinal absorption and metabolism, hepatic transport and metabolism at various levels of functional organization, etc.) and toxicodynamic processes (molecular, biochemical and physiological adversity-related mechanisms) have been and are being developed (see, e.g., OECD guidelines and the EPA list in agencies’ home sites). Regarding toxicokinetic processes, the *in vitro* skin absorption method is included in OECD guidelines (No. 428) as well as the *in vitro* CYP induction in human hepatocytes which has been validated (Bernasconi et al., 2019). Despite a lack of validation thus far, various physiologically-based kinetic (PBK) models are used as an integral part of hazard and risk assessment of pesticides and their metabolites (Paini et al., 2019). Regarding toxicodynamic processes, most OECD-approved guidelines are within the endocrine disruptor screening area (e.g., No. 455, 456, 458, 493). Non-validated *in vitro* tests are increasingly being used for providing information that can be used as part of the weight of scientific evidence in characterizing mechanism or mode of action or a hazard to better understand the toxicity observed with parent (or lead compound(s) in QSAR and read across) and metabolite(s) and used in risk-based decision making (e.g., Miccoli et al. (2022)). Furthermore, the results of these *in vitro* methods are important in developing adverse outcome pathways (AOPs) as building blocks for molecular initiating and key events and their relationships, which are useful in the frame of integrated approaches to testing and assessment (IATA) schemes, provided they are scientifically justifiable and suitable for risk assessment purposes. As an example, EFSA published an AOP-informed IATA on developmental neurotoxicity showing the applicability of a Developmental Neurotoxicity (DNT) *in vitro* test battery for hazard identification and characterisation and its use in regulatory decision making (EFSA PPR Panel et al., 2021a).

However, the use of such *in vitro* methods as well as experimental evidence obtained with the approaches mentioned above should be considered on a case-by-case basis and proper justification should always be provided for the use of the alternative approaches.

Many cell-based test panels for target organ toxicities have been developed and many of them are commercially available. Examples of such panels are micro-physiological test systems (Marx et al., 2016), tissue chip technologies (Rusyn et al., 2022), imaging based (cell painting) phenotypic profiling (Nyffeler et al., 2021 and *in vitro* cell stress panels (Hatherell et al., 2020), among many other test systems.

### *In vivo* testing methods

In case the above *in silico* and *in vitro* assessments do not allow for convincing conclusions as to the absence or insignificant probability of human hazard, strategies to test the toxicological relevance of metabolites (EFSA PPR Panel, 2016) constitute a basis to apply appropriate *in vivo* testing methods. Metabolites that are not covered by the toxicological data of the parent compound and demonstrated suspicion of potent toxicity in *in vitro* testing may require the use of *in vivo* animal studies. As a first step, a 28-day oral toxicity study in rats (OECD TG 407) or preferably a 90-day rat study (OECD TG 409) for the metabolite is suggested, using the same strain of laboratory animals and the same experimental conditions as used for the parent. The choice of the study would depend on the toxicological profile of the parent compound and on the study from which the reference dose of the parent compound was derived and should be tailor-made.

### Risk assessment

**FIGURE 4:**
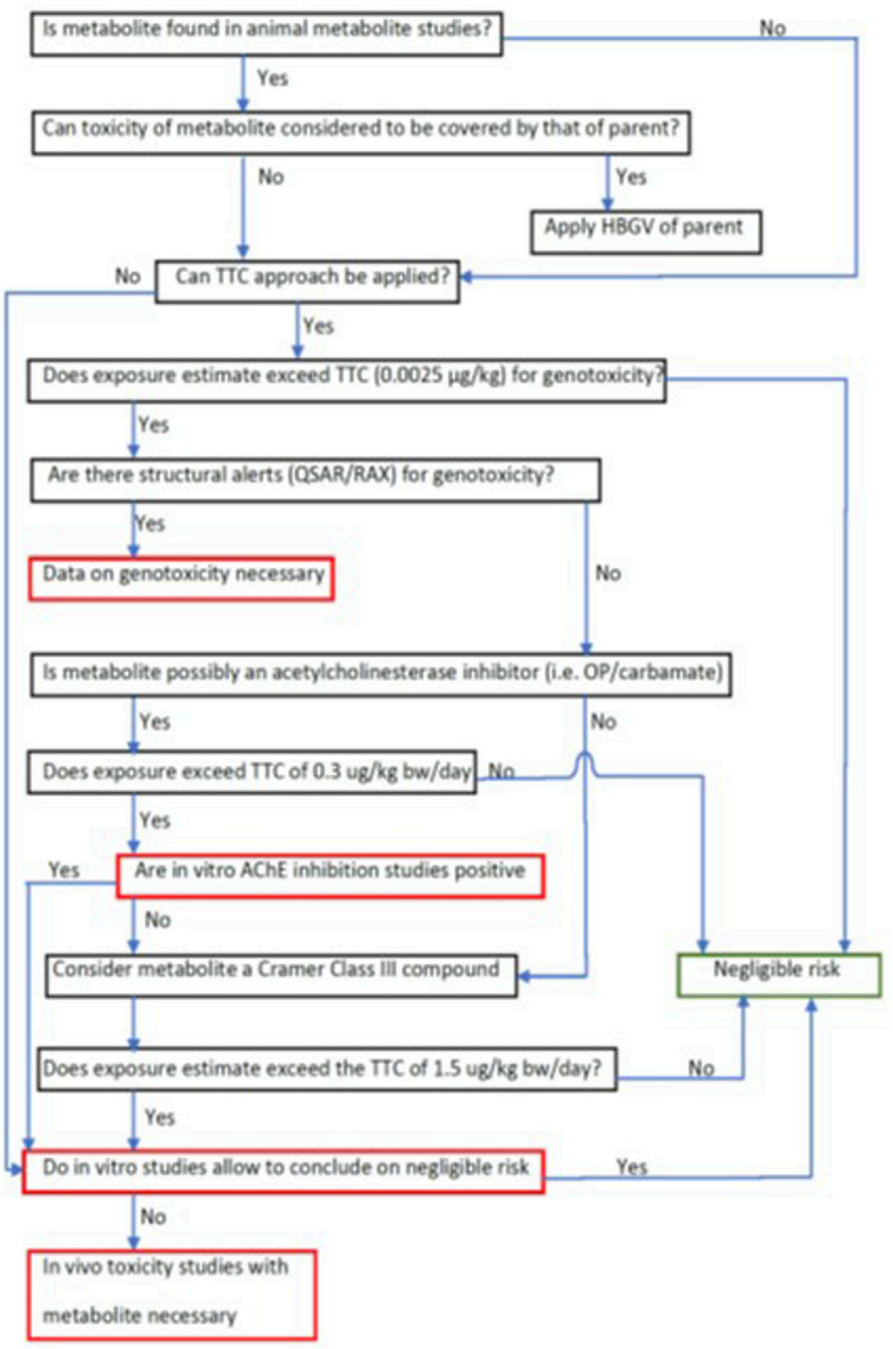
Flow chart: Assessment scheme for (pesticide) metabolite for which no empirical toxicological data are available. Red-framed rectangles contain critical question/decision points.

A weight of evidence approach based on key factors, such as similarity of chemical structure and the nature of the major metabolite, is necessary to establish if the toxicity of a metabolite is covered by the toxicity of the parent compound. If this is the case, the risk assessment is performed by using the reference values of the parent and considering the additional hazard/risk associated with higher exposure to the metabolite. If not, or if specific alerts are detected, targeted toxicity studies may be required, on a case-by-case basis, to better establish the toxicity profile of a metabolite and to enable establishment of reference values (see flow chart Figure 4). EFSA’s Scientific Committee guidance on weight of evidence has proposed a three-step approach where the evidence is assembled, weighted and integrated as well as a summary reporting table (EFSA SC et al., 2017). In data poor situations, the estimated exposure can be weighed against the TTC to consider whether additional data are needed (EFSA SC, 2019).

## Concluding remarks and future research and assessment needs

### Towards 3Rs and non-animal toxicological risk assessment of pesticides

Although animal toxicity studies have been, and still are, considered critical for the risk assessment of pesticides, and they are codified in official regulation and guidances, there has been a constant drive to reduce and even abolish animal testing within the sphere of 3Rs principles (Naik et al., 2022). One of the most important considerations with regards to pesticide metabolites is the production of unique or disproportionate human metabolites vis-à-vis rat *in vivo*/*in vitro* metabolites.

The Scientific Opinion from the EFSA PPR panel describes the actions to be taken to detect such metabolites and as to proceed in case they have been detected. This option will prevail as long as at least the OECD 417 test guideline is enforced and considered useful for risk assessment (EFSA PPR Panel et al., 2021b). The Scientific Opinion considered that, although disproportionate human metabolites are co-tested in toxicity-tested laboratory species, nevertheless they are occurring in *in vitro* systems at lower levels than in human hepatocytes. Therefore, their potential toxicity is considered not properly covered by toxicological studies and a toxicological assessment is needed. The Scientific Opinion recommended for both unique and disproportionate human metabolites the use of a NAM-based approach, as starting point.

### Connections with One Health concept and goals

As widely acknowledged, significant relationships and interactions between environmental stressors and their holistic environmental and biological impact remain mostly uncharacterised. Therefore, approaches to depict metabolism and metabolites and further developments in various analytical and life science techniques, as well as the concepts of multi-stressors in the exposome and multi-receptors in One Health, provide options to better understand the impact of chemical stressors and their metabolites on humans, animal species and the environment (see e.g., Magurany et al., 2023).

The operationalisation of One Health (OH https://www.who.int/health-topics/one-health#tab=tab_1/ https://health.ec.europa.eu/one-health_en) through embracing trans-disciplinary considerations including better understanding of metabolism and toxicity of pesticides will help to deal with growing concerns and threats to humans, animals and plant health interconnected to ecosystems.

### Perspectives on analytical predictivity

For many chemical entities, predictable metabolic transformations, often via conjugation or hydrolysis reactions, can be determined *a priori*. Because pesticide metabolism produces compounds with physicochemical and pharmacological properties that may differ substantially from those of the parent chemical, the analytical properties of the metabolites remain difficult to predict (see, e.g., Kirchmair et al., 2015).

The modelling of separation/retention in LC is an active field of research both for theoretical and practical needs but differences between analytical platforms and experimental conditions make standardization difficult (see e.g., Petersson et al., 2018). Therefore, the most notable development is related to the intelligent screening of the MS data obtained. Computational methods that allow the identification of metabolically labile positions - sites of metabolism - in small organic molecules and the prediction of metabolites will drive the analysis of and high-resolution mass spectrometry (HRMS) data.

Once the chemical structures are putatively determined, the algorithm will take this information into account for rapid deconvolution, peak detection, peak assignment, and integration, allowing a complete mapping and biotransformation of the pesticides to be established (see e.g., Smith et al., 2014). After removing the chemical noise, all detectable MS signals could be extracted, and the putative metabolite lists of the analyte and the data are then compared and combined into a final identification score.

Hence, fundamental advances in the prediction of metabolic sites and metabolites would result from better identification of major and minor products, improved experimental and statistical software tools for the normalisation of HRMS data, integration with transport phenomena, and experimental data on concentrations and reaction rates. Therefore, analytical techniques for the detection and identification of drug metabolites are likely to continue to focus on the use of MS-based detection with increasing level of sophistication and sensitivity for the foreseeable future (e.g., Yuan et al., 2005).

### Integration of *in vitro* kinetic data in physiologically-based kinetic (PBK) models

Relating data on species differences in intrinsic hepatic clearance and/or rate of formation of metabolites obtained from *in vitro* test systems to possible data on species differences in internal exposure (e.g., Cmax or AUC or other relevant TK parameters to interpret toxicity data) is not straightforward. In its scientific opinion, formation of a metabolite by human hepatocytes 4-fold above that in animal hepatocytes was selected by the PPR panel to define a DHM (EFSA PPR Panel et al., 2021b). This does not necessarily mean that with equal exposure to the parent chemical, the internal metabolite concentration in humans would be 4-fold higher compared to that in the *in vivo* test species study. This is particularly relevant since the internal concentration of a chemical is dependent on various kinetic parameters, including absorption, distribution, blood flow to the liver, binding to plasma proteins, excretion, etc. In an ideal situation, data on the most relevant kinetic parameters would be available for the different species and applied in PBK models that allow the description (or prediction) of dose-dependent internal exposure of the parent chemical and the (most relevant) metabolites in the species of interest. It must be noted that the testing approach described in the scientific opinion (EFSA PPR Panel et al., 2021b) was not developed to obtain chemical-specific input parameters for PBK models. It is therefore recommended to develop a harmonised guidance to highlight the requirements for generating reliable chemical-specific input parameters for PBK models and illustrate their use through relevant case studies so that these can be used for regulatory applications as demonstrated recently in the OECD guidance and related case studies (OECD, 2021a, b; Paini et al., 2019; Punt et al., 2023).

### Towards novel hazard and risk assessment paradigms

It is widely recognized by research bodies and regulatory agencies that there is a fundamental need for a paradigm shift from *in vivo* animal experiments to non-animal approaches to employ novel analytical and molecular biological techniques together with *in silico* computational and extrapolation techniques. These needs involve several steps, starting with 1. *in silico* and *in vitro* NAMs and analytics to generate the data to be used for step 2. quantitative *in vitro* to *in vivo* extrapolation (QIVIVE) on the basis of PBK and dynamic modelling including knowledge of ADME properties for the compound under assessment (OECD, 2021). The outcome of these models is critical for step 3. the derivation of PODs/RPs using quantitative data on interspecies/human variability, and finally step 4. the development of a guidance on the use of kinetic data in risk assessment using tiered approaches and applicable in practice to a range of regulatory contexts taking into account problem formulation i.e., question, resources and refinement needs. Several regulatory agencies and international consortiums are developing such paradigms (Paul Friedman et al., 2020; OECD, 2021 a; b; Escher et al., 2022; Cattaneo et al., 2023; Carnesecchi et al., 2023). In this context EFSA has very recently published the TKPlate 1.0 platform which allows predictions of kinetic parameters and concentrations of chemicals in body fluids and organs in humans, test species and farm animals using PBK modelling as well as (Q)IVIVE. Other modules in the platform also allow back-calculation of exposure (reverse dosimetry) from biomonitoring data, internal benchmark dose modelling, dynamic energy budget modelling for species of ecological relevance and mixture risk characterisation (Dorne et al, 2023; Bossier et al., 2023a, Bossier et al., 2023b).

### Integrated approach for estimating metabolism and toxicodynamic markers in a holistic system

Over the last years, extensive research efforts have been ongoing to develop *in vitro* methods to predict the *in vivo* kinetic behaviour for compounds of concern (e.g., Bessems et al., 2014). However, less emphasis has been placed on characterizing *in vitro* methods in which both TK and TD properties, as far as they could be confidently measured, are taken into account together and estimated in the same *in vitro* system. This concept has been promoted by, e.g., the guidance document on Good *In Vitro* Method Practices (GIVIMP) document and some earlier efforts for realizing this goal has been undertaken within the EU Predict-IV project (Kramer et al., 2015). Although *in vitro* systems for measuring metabolism, e.g., hepatocyte-derived systems, have been available for a long time, the idea of combining metabolism and some TD indicators in the same experimental setup, such as metabolomics, have been explored recently (Bowen et al., 2023). Although a few TD *in vitro* systems would harbour necessary metabolising systems, it would be beneficial to develop holistic approaches providing means to measure both TK and TD processes in the same inherently integrated setup.

### ‘Natural’ versus ‘synthetic’ pesticides

There has been a growing trend in recent decades to be more environmentally friendly: in the area of pesticides, this means that the number of ‘natural’ or better ‘biological pesticides’ has increased. These ‘BioPesticides’^2^ include e.g., plant extracts/botanicals (pyrethrin insecticide extracted from certain chrysanthemum plants or azadirachtin, an extract from the neem tree), microbials (bacteria, algae, protozoa viruses, fungi), pheromones and semiochemicals, and macrobials/invertebrates such as insects and nematodes. Obviously, investigations for metabolism and metabolites are relevant and requested for plant extracts and botanicals, with apparent additional difficulties that botanicals are complex mixtures and generally inadequately defined in terms of chemical composition and potential activities. Regarding microbials, a possibility for the organisms to produce secondary metabolites is often dismissed. However, it is still uncertain to what extent these ‘natural’ pesticides would supplant or replace ‘synthetic’ pesticides and how they will be assessed regarding potential toxicity and other problems in use.

## Footnotes

1 Another guideline study OECD503 on metabolism in livestock is used for elucidating residues in livestock, but not primarily for toxicity assessment.

2 Biological pesticides—OECD, see https://www.oecd.org/chemicalsafety/pesticides-biocides/biological-pesticides.htm; accessed on 18.10.2023.
